# Evaluation of retinal microcirculation alterations using OCTA in hyperopic ametropic amblyopia patients before and after treatment

**DOI:** 10.1007/s10792-023-02707-0

**Published:** 2023-04-21

**Authors:** Ting Rao, Wen Zou, Xiaoqin Hu, Hai He, Wei Luo, Zhipeng You

**Affiliations:** 1 Affiliated Eye Hospital of Nanchang University, Nanchang, China; 2 Nanchang Hongdu Hospital of Traditional Chinese Medicine, Nanchang, China

**Keywords:** Hyperopic ametropic amblyopia, Amblyopia treatment, Optical coherence tomography angiography, Vessel density, Perfusion density

## Abstract

**Purpose:**

We aimed to compare retinal microcirculation in hyperopic ametropic amblyopia patients before and after treatment and in healthy children using optical coherence tomography angiography (OCTA), and to explore the pathogenesis of hyperopic ametropic amblyopia.

**Methods:**

Eighteen patients with hyperopic ametropic amblyopia aged 4–8 years were selected as the patient group, and 18 age-matched healthy children were randomly selected as controls. The foveal avascular zone (FAZ) area, perimeter and circularity, vessel density (VD) and perfusion density (PD) of macular superficial retinal capillary plexus, macular thickness, peripapillary retinal nerve fiber layer thickness, and ganglion cell-inner plexiform layer thickness were compared between both groups. After 6 months of amblyopia treatment, the same parameters were measured again.

**Results:**

The VD and PD in the central, inner, inner nasal, and inner inferior regions in hyperopic ametropic amblyopia were lower than in the control group after adjustment for axial length. After 6 months of treatment, the VD increased significantly, except in the outer nasal and outer inferior regions. The PD in the central (*p* < 0.001), inner superior (*p* = 0.001), inner inferior (*p* = 0.011) and inner temporal (*p* = 0.026) regions increased. The FAZ perimeter and circularity significantly differed between the groups. After 6 months of treatment, the FAZ area and perimeter decreased, but circularity increased.

**Conclusion:**

Hyperopic ametropic amblyopia eyes showed a significant decrease in vessel and perfusion densities. After amblyopia treatment, the vessel and perfusion densities of patients with hyperopic ametropic amblyopia increased, suggesting that abnormalities in the microvascular system are a pathogenic factor of amblyopia.

## Introduction

Amblyopia is a serious public health problem that seriously affects the normal development of children’s visual function, and its incidence rate is 2–5% [1]. If the treatment is not timely, the visual damage caused by amblyopia can become permanent [2]. It was formerly believed that amblyopia patients had no organic changes in their eyes. With the development of detection tools, more in-depth research on the eye structure of amblyopia patients was conducted, and it was found that their eye structure differed from that of healthy individuals. Optical coherence tomography angiography (OCTA) provides a detailed view of the microvascular networks, allowing accurate, rapid, and non-invasive quantification [3]. Numerous studies have confirmed the presence of macular microvascular abnormalities in amblyopic eyes [4, 5].

The traditional comprehensive treatment of children with amblyopia currently includes visual acuity training and effective clinical methods [6]. In diagnosing and treating amblyopia, more attention is paid to whether patients’ vision is improved [7–9]. There are still relatively few reports on whether the retinal structure of amblyopia patients has changed accordingly before and after treatment. The retinal capillary network and the microcirculation supply oxygen and nutrients to the retina. The retinal microvascular system is an important basis for maintaining visual function. Therefore, the retinal microcirculation alterations before and after amblyopia treatment will become a new research direction. This study aimed to quantify and compare retinal microcirculation alterations in hyperopic ametropic amblyopia patients before and after treatment, as well as with those of age-matched healthy children, using the OCTA technique.

## Methods

### Subjects

This case–control study was conducted from March 2021 to June 2022 at the Affiliated Eye Hospital of Nanchang University, Nanchang, China. Eighteen patients (36 eyes) with hyperopic ametropic amblyopia aged 4–8 years were selected as the patient group, and 18 age-matched healthy children (36 eyes) were randomly selected as controls. The protocol was approved by the Ethics Committee of the Affiliated Eye Hospital of Nanchang University, and the study was conducted according to the tenets of the Declaration of Helsinki for research involving human subjects. Written informed consent for the examinations was obtained from the participants or one of their parents/legal guardians, and this was approved by the Ethics Committee. Hyperopic ametropic amblyopia was defined as those cases in which the best-corrected visual acuity in both eyes caused by hyperopic ametropia was lower than that corresponding to the age of the patient during the visual development period. Amblyopia patients enrolled in the study satisfied the following conditions simultaneously: (1) the difference in the spherical lens diopter was less than 1.5 D, (2) the diopter was less than 1.00 D in the cylindrical lens, (3) it was their first visit to an ophthalmologist, and they had not been provided with refractive correction or any other treatments, and (4) the spherical equivalent (SE) was more than + 1 diopters (D). Additionally, age-matched healthy subjects without any known systemic or ocular diseases were enrolled as control subjects. Patients who were uncooperative or had mixed amblyopia, severe amblyopia with poor or eccentric fixation, mental retardation, any structural abnormalities of the eye (either congenital or acquired), or systemic diseases were excluded from the study.

### Routine eye examination

Ophthalmologic examinations performed in all subjects included best-corrected visual acuity (BCVA) with LogMAR visual acuity chart, the subjects’ anterior segment and posterior segment with slit lamp biological microscope, cycloplegic refraction (RKT-7700; NIDEK, Gamagori, Japan), axial length (AL) (IOL Master5.5; Carl Zeiss Meditec AG, Jena, Germany), cover test, and extraocular movements.

### OCT and OCTA inspection

OCT images were obtained using a 5000-HD-OCT Angioplex equipment (Carl Zeiss, Meditec, Inc., Dublin, OH, USA). Macular thickness (MT; nine regions defined by the Early Treatment Diabetic Retinopathy Study [ETDRS]), ganglion cell-inner plexiform layer thickness (GCIPLT; superior, superonasal, inferonasal, inferior, inferotemporal, and superotemporal), and peripapillary retinal nerve fiber layer thickness (RNFLT; superior, nasal, inferior, and temporal) were recorded. OCTA images were obtained using the 5000-HD-OCT Angioplex (Carl Zeiss, Meditec, Inc). The OCTA equipment using an algorithm known as OCT microangiography-complex could automatically scan the macular superficial vascular plexus, including vessel density and perfusion density, which was automatically segmented between the internal limiting membrane and the outer boundary of the inner plexiform layer. The macular area was divided into three circles using 6 × 6 mm scan pattern on the retina: central subfield, parafovea, and perifovea. The paracavity and central concave edge were divided into nine regions: inner superior, outer superior, inner nasal, outer nasal, inner inferior, outer inferior, inner temporal, outer temporal, and central. Values for vessel density, perfusion density, foveal avascular zone (FAZ) area (mm^2^), perimeter (mm), and circularity were obtained automatically. Scanning was repeated if the signal strength index quality was < 7/10. All images with significant artifacts or poor quality were excluded from the analysis.

### Follow-up protocol

The two groups of patients were subjected to a regular follow-up examination 6 months after the first visit. Hyperopic ametropic amblyopia patients received amblyopia treatment based on refractive correction. Ophthalmologists designed the amblyopia treatment plan according to the amblyopia condition of each patient, including masking treatment and a visual function training plan. The visual function training content included fine visual work and visual stimulation, such as red flash, black-an-white grating, or color grating with sinusoidal contrast sensitivity change, once a day, 30 times as a course of treatment, followed by outpatient reexamination after one course of treatment. The control participants did not receive any treatment.

### Statistical analyses

Statistical analysis was performed using SPSS, version 25 (IBM Corp., Armonk, NY, USA). Categorical variables are shown as numbers and percentages; quantitative variables are presented as the means ± standard deviations, or medians (interquartile ranges). Variable normality was inspected using the Shapiro–Wilk test for all variables. The Welch two-sample t-test or the Wilcoxon Signed Rank test were performed to compare baseline parameters between the hyperopia ametropic amblyopia eyes and the control eyes. A one-way analysis of covariance (ANCOVA), which was controlled using axial length, was used to evaluate differences in the OCTA parameters between the hyperopia ametropic amblyopia eyes and the control eyes. The paired t-test or the paired Wilcoxon Signed Rank test were performed to compare each parameter before and after treatment. The relationship between VD, PD, FAZ, and BCVA was assessed using linear regression analysis after adjustment for all known confounders. For all analyses, a two-tailed *p* < 0.05 was considered statistically significant.

## Results

### Participant baseline characteristics

The baseline characteristics of the participants are summarized in Table 1. In total, 36 eyes in 18 participants had hyperopic ametropic amblyopia and 36 eyes in 18 control participants were controls. There was no significant difference between the cases and controls regarding age (*p* = 0.087) or sex (*p* = 0.733). However, LogMAR-converted BCVA was significantly worse in the hyperopic ametropic amblyopia cases than in the controls (0.5 vs. 0.0, *p* < 0.001). As expected, SE (*p* < 0.001) and AL (*p* < 0.001) significantly differed between the two groups. After 6 months of treatment, the BCVA (LogMAR) and AL increased in the hyperopic ametropic amblyopia group and showed a significant difference before and after treatment (*p* < 0.001 in both cases). In addition, the SE decreased and showed a significant difference(*p* < 0.001) in the hyperopic ametropic amblyopia group before and after treatment. Correspondingly, the BCVA (LogMAR), AL and SE showed a significant difference between the two groups at the 6 months follow up (*p* < 0.05 in all cases).

**Table 1:** Baseline characteristics of the study participants

	Hyperopic ametropic amblyopia		Control		*P* value		
	Initial	6months	Initial	6months	*P*_*1*_^*c*^	*P*_*2*_^*a*^	*P*_*3*_^*a*^
Age (y)	5.50 (4.00, 7.00)	6.00 (4.50, 7.55)	6.50 (6.00, 8.00)	7.00 (6.50, 8.50)	–	0.087	–
Sex							
*Male*	6.00 (33.33)	6.00 (33.33)	8.00(44.40)	8.00 (44.40)	–	0.733^e^	–
*Female*	12.00 (66.67)	12.00 (66.67)	10 .00(55.60)	10 .00(55.60)			
BCVA (LogMAR)	0.50 (0.40, 0.70)	0.10 (0.00, 0.20)	0.00 (0.00, 0.00)	0.00 (0.00, 0.00)	< 0.001^d^	< 0.001	< 0.001
SE (D)	5.38 (3.75, 8.69)	4.75 (3.25, 8.50)	0.50 (0.25, 0.75)	0.25(0.06, 0.50)	< 0.001^d^	< 0.001	< 0.001
AL (mm)	20.73 ± 0.92	20.93 ± 0.92	22.82 ± 0.71	22.87 ± 0.71	< 0.001	< 0.001^b^	< 0.001^b^

BCVA: best-corrected visual acuity; SE: spherical equivalent; AL: axial length; *P*1, *P* value of hyperopic ametropic amblyopia compared with hyperopic ametropic amblyopia after 6 months treatment; *P*2, *P* value of hyperopic ametropic amblyopia compared with the control group; *P*3, *P* value of hyperopic ametropic amblyopia after 6 months treatment compared with the control group after 6 months follow up

^a^Wilcoxon’s signed rank test

^b^Welch two-sample *t*-test

^c^Paired *t*-test

^d^Paired Wilcoxon signed rank test

^e^Fisher’s exact test

### MT, RNFLT, and GCIPLT

The CMT and ETDRS outer ring thicknesses (superior, nasal, inferior, and temporal) in hyperopic ametropic amblyopia eyes were thicker than those in the control eyes (*p* < 0.05). Interestingly, the CMT of the hypermetropic ametropic amblyopia group after 6 months of treatment was lower than that before treatment (*p* = 0.003). Correspondingly, the ETDRS outer ring thicknesses in hyperopic ametropic amblyopia eyes were thicker than those in the control eyes at the 6 months follow up (*p* < 0.05). Conversely, the CMT at the 6 months follow up showed no significant difference between the two groups (Table 2).

**Table 2:** Comparison of macular thickness between hyperopic ametropic amblyopia and control

	Hyperopic ametropic amblyopia		Control		*P* value		
	Initial	6months	Initial	6months	*P*_*1*_^*c*^	*P*_*2*_^*a*^	*P*_*3*_^*a*^
*MT(µm)*							
CMT	239.86 ± 16.64	232.86 ± 17.07	232.75 ± 10.33	233.03 ± 11.35	0.003	0.033^b^	0.828^b^
AMT	287.00 (272.25, 292.50)	282.50 (274.00, 291.00)	276.00 (271.25, 286.25)	277.00(272.25, 282.75)	0.592^d^	0.074	0.050
*ETDRS inner ring thickness*							
superior	308.30 ± 15.05	306.86 ± 13.22	307.94 ± 16.39	308.92 ± 19.71	0.837	0.923^b^	0.605^b^
nasal	313.50 ± 16.67	312.64 ± 15.67	312.25 ± 14.38	314.19 ± 14.81	0.775	0.734^b^	0.666^b^
inferior	307.00 (294.50, 316.00)	306.00 (296.00, 319.50)	305.00 (296.00, 313.50)	311.00(298.50, 320.00)	0.260	0.774	0.569
tempora	301.00 (284.00, 304.75)	300.00 (288.25, 313.00)	301.00 (287.50, 309.75)	300.67(290.50, 309.00)	0.089	0.382	0.996
*ETDRS outer ring thickness*							
superior	294.50 (281.00, 311.25)	291.00 (281.25, 301.50)	281.00 (281.00, 311.25)	282.50(271.50, 291.00)	0.424	0.017	0.015
nasal	308.00 (299.00, 317.75)	301.50 (292.00, 317.50)	296.00 (287.75, 303.75)	295.00(285.50, 303.00)	0.682^d^	0.007	0.011
inferior	280.00 (264.25, 290.50)	273.00 (262.00, 287.00)	267.00 (261.25, 273.00)	264.50(257.00,272.75)	0.169	0.012	0.047
temporal	271.50 (261.75, 281.75)	272.50 (258.50, 286.75)	260.50 (255.50, 273.75)	260.50(252.00, 271.00)	0.846	0.008	0.009

Abbreviations: CMT, central macular thickness; AMT, average macular thickness; *P*1, *P* value of hyperopic ametropic amblyopia compared with hyperopic ametropic amblyopia after 6 months treatment; *P*2, *P* value of hyperopic ametropic amblyopia compared with the control group; *P*3, *P* value of hyperopic ametropic amblyopia after 6 months treatment compared with the control group after 6 months follow up

^a^Wilcoxon signed rank test

^b^Welch two-sample *t*-test

^c^Paired *t*-test

^d^Paired Wilcoxon signed rank test

The average RNFLT values in the superior and inferior regions significantly differed between the hyperopic ametropic amblyopia and control groups (*p* < 0.05 in all cases). After 6 months of treatment, the average value of RNFLT and the values in the inferior region decreased and were significantly different in the hyperopia ametropic amblyopia group before and after treatment (*p* = 0.017 and *p* = 0.002, respectively). GCIPLT showed no significant differences between the hyperopic ametropic amblyopia and the control group in the average, minimum, superior, inferior, or nasal regions (*p* > 0.05 in all cases).

After 6 months of treatment, the average value of GCIPLT showed no significant difference in the hyperopia ametropic amblyopia group before and after treatment, even though the average value of GCIPLT decreased. At the 6 months follow-up, there was no significant difference between the hyperopic ametropic amblyopia and the control group regarding GCIPLT (p > 0.05 in all cases) or RNFLT, with the exception of the inferior region (*p* = 0.014) (Table 3).

**Table 3:** Comparison of RNFL thickness and GCIPL thickness between hyperopic ametropic amblyopia and control

	Hyperopic ametropic amblyopia		Control		*P* value		
	Initial	6months	Initial	6months	*P*_*1*_^*c*^	*P*_*2*_^*a*^	*P*_*3*_^*a*^
*RNFLT (µm)*							
Average	116.50 (102.25, 119.00)	106.00 (99.50, 112.00)	103.00 (98.00, 106.00)	101.50(97.25, 112.00)	0.017	< 0.001	0.467
Superior	142.00 (133.25, 152.50)	135.00 (121.50, 142.75)	127.50 (121.00, 137. 00)	126.00(114.25, 152. 25)	0.077	< 0.001	0.171
Nasal	67.50 (61.00, 77.75)	69.50 (62.00, 79.00)	65.50 (62.25, 78.75)	64.00(60.00, 72. 00)	0.356	0.735	0.092
Inferior	163.00 (144.00, 172.50)	145.50 (135.25, 158.00)	137.00 (131.50, 143.50)	135.5(117.00, 148. 25)	0.002^d^	< 0.001	0.014
Temporal	75.00 (71.25, 85.00)	75.00 (66.25, 81.00)	71.00 (68.00, 78.00)	70.00(65.00, 81. 00)	0.139	0.069	0.471
*GCIPLT (µm)*							
Average	86.00 (80.25, 89.75)	85.50 (83.25, 91.75)	86.00 (85.00, 88.75)	86.00(83.00, 91.75)	0.086^d^	0.631	0.901
Minimum	78.50 (75.00, 83.00)	83.00(76.00, 87.75)	82.00 (75.25, 84.00)	82.50(75.75, 86.00)	0.087^d^	0.223	0.672
Superior	87.00 (83.00, 91.75)	88.00 (85.00, 93.00)	87.50 (83.50, 89.75)	88.00(85.00, 93.50)	0.325^d^	0.808	0.623
Superonasal	89.50 (86.00, 92.00)	90.00 (87.25, 95.50)	89.50 (86.25, 92.75)	89.00(86.25, 92.75)	0.064^d^	0.764	0.333
Inferonasal	87.11 ± 7.32	87.47 ± 7.52	86.56 ± 4.42	86.81 ± 7.24	0.779	0.698^b^	0.703^b^
Inferior	85.50 (78.25, 89.00)	86.00 (81.00, 91.00)	83.00 (81.00, 86.00)	85.00(79.25, 89.00)	0.281^d^	0.318	0.355
Inferotemporal	83.11 ± 6.53	84.36 ± 7.59	85.42 ± 3.83	85.14 ± 7.22	0.341	0.073^b^	0.355^b^
Superotemporal	84.00 (80.50, 86.00)	85.00 (82.00, 90.75)	85.00 (83.00, 89.00)	85.00(83.00, 89.75))	0.067^d^	0.131	0.830

GCIPLT: ganglion cell-inner plexiform layer thickness; RNFLT: peripapillary retinal nerve fiber layer thickness. *P*1, *P* value of hyperopic ametropic amblyopia compared with hyperopic ametropic amblyopia after 6 months treatment; *P*2, *P* value of hyperopic ametropic amblyopia compared with the control group; *P*3, *P* value of hyperopic ametropic amblyopia after 6 months treatment compared with the control group after 6 months follow up

^a^Wilcoxon signed rank test

^b^Welch two-sample *t*-test

^c^Paired *t*-test

^d^Paired Wilcoxon signed rank test

### VD, PD, and FAZ

**Fig. 1:**
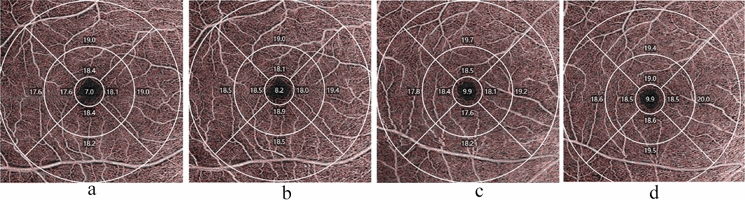
Analysis of optical coherence tomography angiography image. **a** vessel density (VD) of the superficial capillary plexus (SCP) in hyperopic ametropic amblyopia. **b** VD of SCP in hyperopic ametropic amblyopia after 6 months of treatment. **c** VD of SCP in a control eye. **d** VD of SCP in a control eye after 6 months follow up

**Fig. 2:**
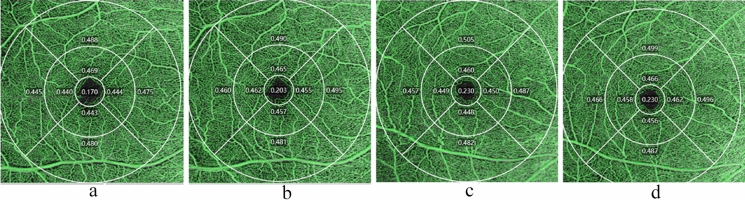
Analysis of optical coherence tomography angiography image. **a** perfusion density (PD) of the superficial capillary plexus (SCP) in hyperopic ametropic amblyopia. **b** PD of SCP in hyperopic ametropic amblyopia after 6 months of treatment. **c** PD of SCP in a control eye. **d** PD of SCP in a control eye after 6 months follow up

**Fig. 3:**
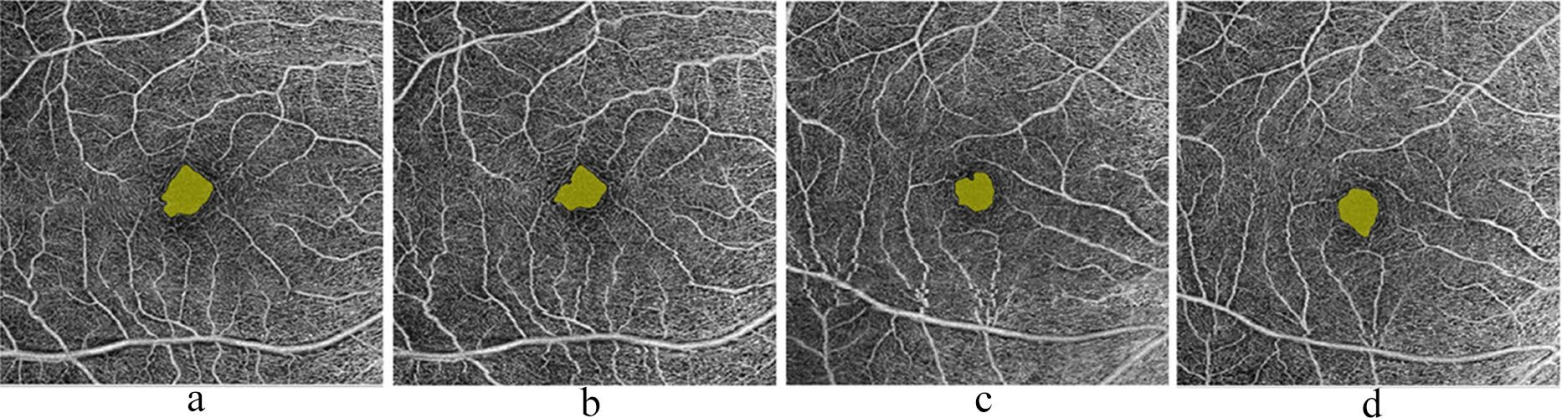
Analysis of optical coherence tomography angiography image. **a** Foveal avascular zone (FAZ) area in the superficial capillary plexus (SCP) in hyperopic ametropic amblyopia. **b** FAZ area in SCP in hyperopic ametropic amblyopia after 6 months of treatment. **c** FAZ area in SCP in a control eye. **d** FAZ area in SCP in a control eye after 6 months follow up

After adjustment for axial length, the VD and PD of the macular superficial capillary plexus (SCP) in the hyperopic ametropic amblyopia group was significantly lower than that in the control group in the central, inner, inner nasal, and inner inferior regions (*p* < 0.05 in all cases). The FAZ perimeter, and circularity significantly differed between the hyperopic ametropic amblyopia and the control group (*p* = 0.012 and *p* = 0.001, respectively). After 6 months of treatment, the VD in the central, inner, outer, full, inner superior, inner nasal, inner inferior, inner temporal, outer superior, and outer temporal regions increased in the hyperopic ametropic amblyopia group and showed a significant difference before and after treatment (*p* < 0.05 in all cases, Fig. 1). There was, however, no significant difference in the outer nasal and outer inferior regions in the hyperopic ametropic amblyopia group before and after treatment (*p* = 0.057 and *p* = 0.235, respectively). The PD in the central (*p* < 0.001), inner superior (*p* = 0.001), inner inferior (*p* = 0.011) and inner temporal (*p* = 0.026) regions increased and showed a significant difference in the hyperopic ametropic amblyopia group before and after treatment (Fig. 2). The FAZ area and perimeter decreased, but circularity increased and significantly differed in the hyperopic ametropic amblyopia group before and after treatment (*p* < 0.05 in all cases, Fig. 3). Interestingly, at the 6 months follow up, there was no significant difference between the hyperopic ametropic amblyopia and the control group regarding VD in the central, inner, outer, full, inner superior, inner inferior, outer superior, outer nasal or outer temporal regions, or regarding PD in the central, inner superior, inner inferior, outer superior or outer nasal regions (*p* > 0.05 in all cases). In addition, the FAZ area and perimeter showed no significant differences between the two groups at the 6 months follow-up (Table 4).

**Table 4:** Comparison of OCTA parameters between hyperopic ametropic amblyopia and control

	Hyperopic ametropic amblyopia		Control		*P* value		
	Initial	6 months	Initial	6 months	*P*_*1*_^*a*^	*P*_*2*_^*c*^	*P*_*3*_^*c*^
*VD (mm*^*−1*^*)*							
Central	7.27 ± 1.94	9.46 ± 1.55	10.40 ± 1.93	10.72 ± 1.94	< 0.001^b^	0.020	0.094
Inner	17.48 ± 0.94	18.41 ± 0.84	18.67 ± 0.74	18.76 ± 0.55	< 0.001	0.001	0.074
Outer	18.41 ± 0.86	18.86 ± 0.46	18.99 ± 0.74	19.09 ± 0.49	0.011	0.617	0.137
Full	17.89 ± 0.79	18.55 ± 0.44	18.68 ± 0.68	18.75 ± 0.46	< 0.001	0.134	0.922
Inner superior	17.54 ± 1.99	18.49 ± 1.02	18.76 ± 0.85	18.75 ± 0.64	0.005	0.208	0.143
Inner nasal	17.80 ± 0.90	18.29 ± 0.98	18.87 ± 0.68	18.88 ± 0.75	0.017	0.002	0.039
Inner inferior	17.20 ± 1.88	18.28 ± 1.01	18.62 ± 1.10	18.64 ± 1.04	0.008	0.003	0.122
Inner temporal	17.47 ± 1.02	18.19 ± 1.03	18.45 ± 1.22	18.65 ± 0.59	0.009^b^	0.057	0.036
Outer superior	18.26 ± 1.41	18.77 ± 0.81	19.02 ± 0.51	19.09 ± 0.59	0.003	0.691	0.477
Outer nasal	19.36 ± 0.83	19.60 ± 0.63	19.98 ± 0.93	20.08 ± 1.23	0.057	0.133	0.062
Outer inferior	18.77 ± 0.94	19.00 ± 0.58	19.17 ± 0.71	19.19 ± 0.62	0.235^b^	0.903	0.018
Outer temporal	17.29 ± 1.46	18.18 ± 0.66	17.80 ± 1.90	17.99 ± 1.96	< 0.001	0.964	0.508
*PD*							
Central	0.17 ± 0.05	0.22 ± 0.04	0.24 ± 0.05	0.24 ± 0.05	< 0.001^b^	0.022	0.082
Inner	0.43 ± 0.03	0.43 ± 0.03	0.45 ± 0.02	0.45 ± 0.01	0.063^b^	0.016	0.014
Outer	0.47 ± 0.02	0.47 ± 0.03	0.47 ± 0.02	0.48 ± 0.01	0.712	0.648	0.003
Full	0.45 ± 0.02	0.45 ± 0.03	0.46 ± 0.02	0.46 ± 0.01	0.313^b^	0.120	0.001
Inner superior	0.43 ± 0.06	0.46 ± 0.02	0.45 ± 0.02	0.46 ± 0.02	0.001	0.125	0.411
Inner nasal	0.43 ± 0.03	0.43 ± 0.04	0.45 ± 0.02	0.45 ± 0.02	0.687^b^	0.040	0.001
Inner inferior	0.42 ± 0.05	0.45 ± 0.02	0.45 ± 0.03	0.45 ± 0.03	0.011	0.016	0.568
Inner temporal	0.43 ± 0.03	0.44 ± 0.02	0.44 ± 0.03	0.45 ± 0.02	0.026^b^	0.418	0.026
Outer superior	0.46 ± 0.04	0.47 ± 0.02	0.48 ± 0.01	0.48 ± 0.01	0.164	0.292	0.052
Outer nasal	0.49 ± 0.02	0.47 ± 0.06	0.49 ± 0.02	0.49 ± 0.03	0.461	0.944	0.089
Outer inferior	0.48 ± 0.02	0.48 ± 0.03	0.48 ± 0.02	0.49 ± 0.01	0.981^b^	0.737	0.013
Outer temporal	0.43 ± 0.04	0.43 ± 0.04	0.44 ± 0.04	0.45 ± 0.02	0.403^b^	0.418	0.038
*FAZ*							
Area (mm^2^)	0.34 ± 0.08	0.25 ± 0.11	0.25 ± 0.07	0.26 ± 0.10	0.002	0.249	0.160
Perimeter (mm)	2.53 ± 0.46	2.00 ± 0.47	2.00 ± 0.28	2.00 ± 0.43	< 0.001	0.012	0.408
Circularity	0.67 ± 0.11	0.75 ± 0.06	0.78 ± 0.05	0.78 ± 0.05	< 0.001	0.001	0.026

OCTA: optical coherence tomography angiography; VD: vessel density; PD: perfusion density; FAZ: foveal avascular zone. *P*1, *P* value of hyperopic ametropic amblyopia compared with hyperopic ametropic amblyopia after 6 months treatment; *P*2, *P* value of hyperopic ametropic amblyopia compared with the control group; *P*3, *P* value of hyperopic ametropic amblyopia after 6 months treatment compared with the control group after 6 months follow up

^a^Paired Wilcoxon signed rank test

^b^Paired *t*-test

^c^A one-way analysis of covariance (ANCOVA)

### Correlation analysis

Poorer BCVA was correlated with reduced PD in the inner region (*p* = 0.012) after adjustment for all known confounders (including sex, age, SE and AL). BCVA was not associated with FAZ area (*p* = 0.403), FAZ perimeter (*p* = 0.489), FAZ circularity (*p* = 0.900) or VD in the central, inner, outer, and full regions (*p* > 0.05 in all cases, Table 5).

**Table 5:** Linear regression analysis between VD, PD, FAZ area, FAZ perimeter, and FAZ circularity with best-corrected visual acuity

	B	Standardized B	*P-*value for linear regression
*VD*			
Central	− 0.174	− 1.480	0.292
Inner	0.596	2.458	0.056
Outer	0.069	0.260	0.840
Full	− 0.435	− 1.498	0.392
*PD*			
Central	7.541	1.577	0.279
Inner	− 24.249	− 3.005	0.012
Outer	− 9.935	− 0.879	0.253
Full	20.021	1.728	0.084
*FAZ*			
Area ( mm^2^)	1.468	0.531	0.403
Perimeter (mm)	− 0.229	− 0.458	0.489
Circularity	− 0.108	− 0.052	0.900

OCTA: optical coherence tomography angiography; VD: vessel density; PD: perfusion density; FAZ: foveal avascular zone

## Discussion

Hyperopic ametropic amblyopia is a common type of ametropic amblyopia; it is mainly due to the lack of visual stimulation in the fovea caused by form deprivation. The mechanism of amblyopia formation is very complex. Lu et al. [10] identified morphological abnormalities in the occipital cortex and in the temporal and frontal cortex, which are projection fields of the visual cortex important for the processing of visual form and object location information, and reported disrupted structural covariance of the visual cortex with other brain regions in amblyopia patients. Some authors believe that the central factors leading to amblyopia formation are the transfer of the dominant column of the eye in the visual cortex, the synaptic structure between neurons, and the degenerative changes of the neurons in the lateral geniculate body [11]. Different authors have studied and analyzed the structural changes of the retina in amblyopia patients, and their views are different [12–15]. Amblyopia often leads to severe visual impairment in patients. The best treatment plan is specified according to the severity of the amblyopia. In general, the BCVA of most amblyopia patients improves after refractive correction treatment, masking treatment, and a training plan for visual function. Whether the retinal structure and the retinal microcirculation of amblyopia patients also have corresponding changes needs to be discussed and studied. Many researchers have applied OCTA to the macula of amblyopia patients to explore whether there are differences in retinal structure and microcirculation [16–19]. However, there are few reports on whether the retinal structure and microcirculation of amblyopia patients exhibit changes after amblyopia treatment. This study not only compared the retinal microcirculation of patients with hyperopic ametropic amblyopia, but also included a longitudinal comparison to explore the retinal microcirculation of patients with hyperopic ametropic amblyopia after 6 months of amblyopia treatment.

In this study, we noted a significant decrease in VD and PD in the central, inner, inner nasal, and inner inferior regions in hyperopic ametropic amblyopia eyes compared with those in control eyes. Furthermore, we found a decrease in FAZ circularity and an increase in the FAZ perimeter in hyperopic ametropic amblyopia eyes compared with those in control eyes. The CMT was thicker than in control eyes, while there was no significant difference in peripapillary RNFLT and GCIPLT. The results of the linear regression analysis helped us determine the positive association between PD in the inner region and BCVA after adjustment for all known confounders. The vascular plexus in the superficial macular area supplies most of the nutrition for the retinal nerve fiber layer, ganglion cell layer, and inner core layer [20]. In the amblyopia group, the VD and PD in the superficial macular area decreased, affecting retinal function and leading to poor vision. After amblyopia treatment, BCVA improved, the VD and PD in the superficial macular area of the corresponding area increased, and the CMT decreased. As a consequence, the better image quality promoted the development of retinal microvessels in the macular region of amblyopia-affected eyes and ameliorated the development of ischemia in the retinal fovea. We inferred that the low in VD and PD in the superficial macular region is due to the difficulty of clear imaging of the retina of hyperopia ametropic amblyopia patients. Therefore, the abnormal visual stimulation leads to stagnation of the development of retinal microvessels in the macular region of amblyopia-affected eyes, the development of ischemia in the retinal fovea, a low efficiency of blood distribution in the retinal branch network, and a widening of the retinal vessels. Regarding the MT thickening observed in amblyopic eyes, we believe that the unclear imaging and the reduction of effective light stimulation may affect the process of RGC reduction, hinder the degeneration of the retina and the formation of macular depression, and facilitate retinal thickness. This also confirms why some children with amblyopia still cannot improve their visual acuity despite receiving standardized amblyopia treatment [21]. Lonngi et al. [22] and Cinar et al. [23] reported that the macular vessel density (VD) of the SCP was lower in the amblyopic group than in the control group. These reports are consistent with the results of our study. Although no significant vascular damage was demonstrated by OCTA in amblyopic eyes, localized defects may be specific to it. However, Demirayak et al. [24] showed that there were no differences between amblyopic eyes, controls, and fellow eyes of patients with unilateral amblyopia in the VD of the SCP and deep capillary plexus visualized by OCTA. We believe that the differences in the results reported in the literature are mainly associated with the following aspects: first, the heterogeneity in the depth and type of amblyopia (strabismic, anisometropic, or mixed amblyopia) might have influenced the data. Second, most of the previous studies did not require participants to no have been treated previously, and the specific time when they were diagnosed with amblyopia was not reported. In contrast, the patients selected for this study only included children with hypermetropic ametropic amblyopia who visited the hospital for the first time, without any previous treatment, and the control group was composed of age-matched participants to minimize the impact of age, amblyopia type, and other sources of heterogeneity in the data. In addition, our study showed that VD, PD, and BCVA significantly improved after amblyopia treatment, which further confirms that the abnormalities in the retinal microvascular system were the pathogenic factor of amblyopia. However, there are some limitations to this study: (1) the follow-up time of amblyopia treatment was short, and in some patients a total cure of the condition was not achieved. Whether the length of the amblyopia period affects the retinal blood flow density and retinal thickness in the macular region or not was not explored; (2) The OCTA technology available at our hospital can only detect the density of macular superficial retinal capillary plexus, but cannot measure the macular perfusion density of the deep capillary plexus, which limits the results of the study; (3) The sample size was not large enough, and should be expanded in future studies to further explore the interaction mechanism between retinal microcirculation changes and clinical efficacy in patients with hyperopia ametropic amblyopia.

## Conclusions

The retinal microcirculation of hyperopic ametropic amblyopia patients is different from that of age-matched children, and is improved after amblyopia treatment. This suggests that abnormal retinal microcirculation is one of the pathogenic factors underlying amblyopia, which could be useful in the prevention and treatment of this ophthalmic syndrome.

## Data Availability

The data that support the findings of this study are available from the corresponding author upon reasonable request.
